# Monthly direct and indirect greenhouse gases emissions from household consumption in the major Japanese cities

**DOI:** 10.1038/s41597-021-01086-4

**Published:** 2021-11-23

**Authors:** Yin Long, Yida Jiang, Peipei Chen, Yoshikuni Yoshida, Ayyoob Sharifi, Alexandros Gasparatos, Yi Wu, Keiichiro Kanemoto, Yosuke Shigetomi, Dabo Guan

**Affiliations:** 1 Graduate School of Engineering, University of Tokyo, 7-3-1 Hongo, Bunkyo-ku, Tokyo, 113-8654 Japan; 2 Graduate Program in Sustainability Science - Global Leadership Initiative, The University of Tokyo, 5-1-5 Kashiwanoha, Kashiwa, Chiba, 277-8563 Japan; 3 The Bartlett School of Sustainable Construction, University College London, London, WC1E 7HB UK; 4 Graduate School of Humanities and Social Science. 1–31- Kagamiyama, Hiroshima University, Higashi, Hiroshima, 739-8530 Japan; 5 Institute for Future Initiatives, University of Tokyo, 7-3-1 Hongo, Bunkyo-ku, Tokyo, 113-8654 Japan; 6 Institute for the Advanced Study of Sustainability (UNU-IAS), United Nations University, 5-53- Jingumae, Shibuya-ku, Tokyo, 150-8925 Japan; 7 Research Institute for Humanity and Nature, 457-4 Motoyama, Kamigamo, Kita-ku, Kyoto 603-8047 Japan, Kyoto, Japan; 8 Faculty of Environmental Science, Nagasaki University, 1-14 Bunkyo-machi, Nagasaki, 852-8521, Japan, Nagasaki University, Nagasaki, Japan; 9 Department of Earth System Science, Tsinghua University, Beijing, 100084 China

**Keywords:** Climate-change mitigation, Environmental impact, Sustainability

## Abstract

Urban household consumption contributes substantially to global greenhouse gases (GHGs) emissions. Urban household emissions encompass both direct and indirect emissions, with the former associated with the direct use of fossil fuels and the latter with the emissions embodied in the consumed goods and services. However, there is a lack of consistent and comprehensive datasets outlining in great detail emissions from urban household consumption. To bridge this data gap, we construct an emission inventory of urban household emissions for 52 major cities in Japan that covers around 500 emission categories. The dataset spans from January 2011 to December 2015 and contains 12,384 data records for direct emissions and 1,543,128 records for indirect emissions. Direct emission intensity is provided in g-CO_2_/JPY to facilitate both future studies of household emission in Japan, as well as act as a reference for the development of detailed household emission inventories in other countries.

## Abstract (summary)

**Table:** 

Measurement(s)	carbon dioxide emission
Technology Type(s)	Consumption of Goods
Factor Type(s)	urban carbon footprint
Sample Characteristic - Environment	city
Sample Characteristic - Location	Japan

Machine-accessible metadata file describing the reported data: 10.6084/m9.figshare.15188058

## Background & Summary

Cities currently account for the bulk of the ever-increasing greenhouse gas (GHG) emissions globally^1^, with large variability between countries and regions^2^. Further to the increasing levels of GHG emissions from direct energy use in cities, urban household consumption has emerged as an even greater (and often hidden) source of emissions. For example, the GHG emissions embodied in household consumption can account for as much as 70–80% of the total final emissions for the world’s three largest (and highly urbanized) economies, namely USA^3,4^, China^5–9^, and Japan^10,11^. Furthermore, studies have pointed that urban household consumption contributes to more than 70% of GHG emissions from cities^5,12^, accounting for a large fraction of total national emissions^13^.

Reducing the carbon footprint of urban household consumption has been identified as one of the most promising climate change mitigation strategies. For example, in some developed countries such as Japan, it could reduce nearly 40% of national GHG emission by 2030^14^. However, estimating the GHG emissions of urban households is rather complicated.

To start, the field of urban carbon accounting is still nascent. Major improvements would be needed before obtaining more accurate and consistent results considering that cities are open systems that continuously interact with their hinterlands^15^. Accounting for direct GHG emissions has traditionally been conducted at the national level using energy use data or at the global level based on satellite observations. Several well-established databases currently provide estimates of global emissions at somewhat granular spatial and temporal scales, e.g. the Open‐source Data Inventory for Anthropogenic CO_2_ (ODIAC)^16^, and the Emission Database for Global Atmospheric Research (EDGAR)^17^. Top-down approaches that employ atmospheric inversion techniques can estimate urban GHG fluxes and concentrations^18,19^. Downscaling methods using proxies such as population density and commercial activity have been used to estimate city-level GHG emissions using total global or national emissions data available in the above-mentioned databases^2^.

Using combinations of the above-mentioned approaches, some efforts have been made to estimate urban GHG emissions at city level^20,21^. Globally, some of the more noteworthy developments have been city-level emission datasets using data compiled by organizations such as the Carbon Disclosure Project (CDP: https://www.cdp.net/en) and the Carbon Climate Registry (https://carbonn.org/)^22^ and the China Emission Accounts and Datasets (CEADs: https://www.ceads.net/). However, some scholars have expressed major concerns about the reliability of urban emission datasets that are based on data self-reported from city governments and other similar organizations, as studies have found that such inventories sometimes omit certain fuels and types of emissions, while they estimate transport-related emissions differently^23^.

Households, and their consumption, pose quite severe complications for city-level GHG accounting, and are rarely if ever included in city-level GHG emission datasets such as the ones mentioned above. This is because household emissions are very diverse, ranging from emissions associated with direct fuel use for household heating, cooking, and transport (i.e. direct emissions), to emissions embodied to the multitude of goods (e.g. food, durable goods) and services (e.g. entertainment) (i.e. indirect emissions) that urban household consumes^24–26^. Top-down approaches such as the ones mentioned above cannot be mobilized effectively to capture household-level emissions considering the significant component of indirect emissions, while downscaling approaches become particularly uncertain due to the large national heterogeneities^27,28^.

Methodologically, recent developments in the field of environmental extended input-output modelling have been increasingly used to estimate the indirect emission component of household consumption in countries such as China^5,7,26^, India^24,29^, and Japan^13,30,31^. This emerging literature has explored diverse topics such as the aggregate emissions of households^32–34^, single-city case studies^35–37^, multi-city case studies^38,39^, or even specific phenomena such as the inequality of GHG emissions across houseolds^40,41^. However, such techniques require the careful combination and cross-mapping of consumption inventories and input-output tables in order to provide reliable estimates of indirect emissions. Although the consumption inventories usually define the level of detail of the matching process, input-output tables tend to contain fewer sectors than inventories.

The above suggests that city-level GHG emission accounting is still not well developed and lags behind national-level accounting. This is due to various issues associated with resource intensiveness, and limited access to comprehensiveness and verifiable data, especially related to indirect household emissions. Currently, there is a scarcity of multi-city databases that quantify both direct and indirect emissions in a comprehensive and consistent quality manner. Apart from the methodological constraints outlined above, this is compounded by the very diverse capability of different cities to collect data.

Against this background, this Data Descriptor provides a comprehensive and consistent database of the direct and indirect emissions of urban households in the major Japanese cities. The database created contains several hundred emission categories for the period January 2011 to December 2015 based on the monthly consumer expenditure survey and input-output lifecycle inventory. To our knowledge, both are the finest-scale open data currently available in Japan cognizant of the timespan, number of commodities, and free availability. (see Limitations and Acknowledgement). The proposed Data Descriptor is therefore the most comprehensive database of urban household emissions for Japan (i.e. 12,384 records and 1,538,136 records for direct and indirect emissions, respectively).

## Methods

### Dataset scope

**Fig. 1:**
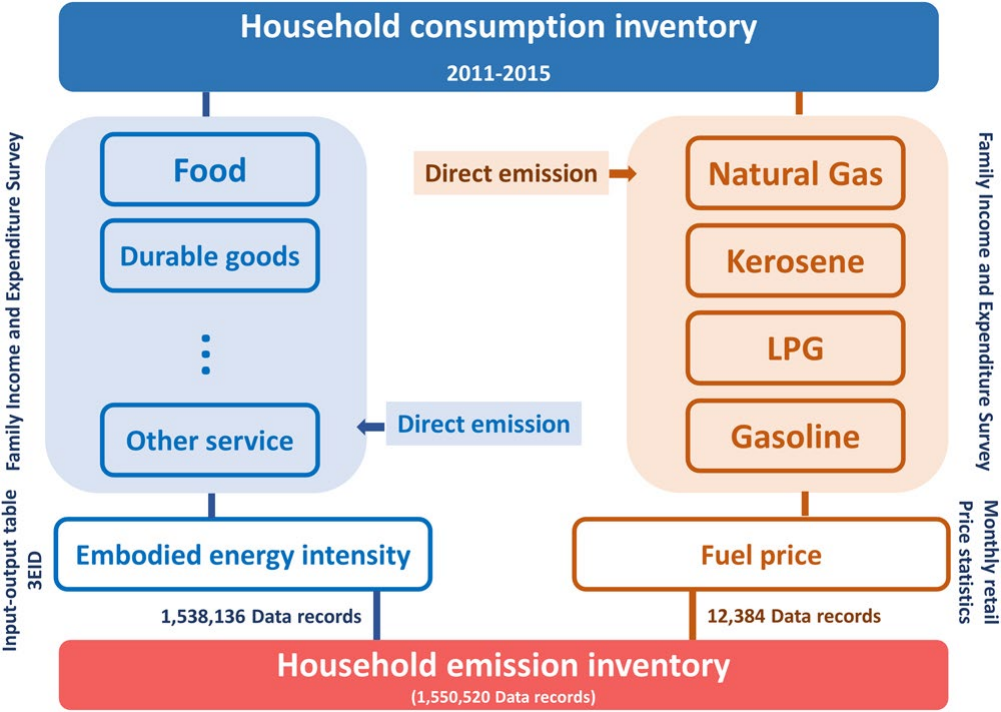
Structure of the dataset.

The dataset outlined in this Data Descriptor contains the monthly direct and indirect emissions of urban households in major Japanese cities for the period between 2011 and 2015 (Fig. 1). The basis of the dataset is the monthly “Family Income and Expenditure Survey” (FIES) collected by the Statistics Bureau of Japan^42^. This survey quantifies in a consistent manner the expenditures of Japanese households for around 500 distinct categories of goods and services. The consumption data is coupled with the 2011 and 2015 databases of the Embodied Energy and Emission Intensity Data for Japan Using Input-Output Tables (3EID)^43–46^.

**Fig. 2:**
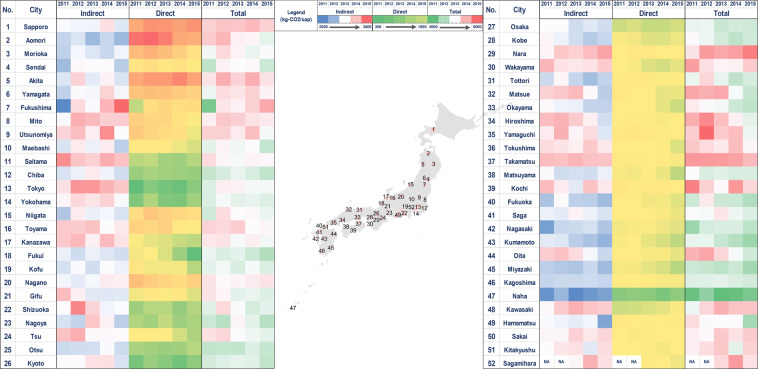
Average monthly direct and indirect emissions by city and year.

This results in a unique dataset that quantifies consistently over time and across different cities the direct and indirect GHG emissions of urban households. The direct emissions are due to the direct combustion of fossil fuels in housing, transport, and other activities (see “Direct Emissions” below) (Fig. 1). The indirect emissions reflect the emissions embodied in approximately 500 categories of goods and services consumed by urban households (see “Indirect Emissions”) (Fig. 2).

The spatial scope of the dataset is the major Japanese cities: 51 major cities (2011 and 2012 data), or 52 major cities (2013, 2014, and 2015 data). These cities encompass all of the capitals of the 47 prefectures, as well as four major cities that are not prefectural capitals, namely Kawasaki (Kanagawa Prefecture), Sagamihara (Kanagawa Prefecture, from 2013 to 2015 data), Hamamatsu (Shizuoka Prefecture), Sakai (Osaka Prefecture), and Kitakyushu (Fukuoka Prefecture).

The files of the different annual FIES are first processed in order to extract the relevant parts of data for both direct and indirect emission calculation. The following sub-sections include more information about the direct and indirect emissions included in the dataset, as well as its limitations.

### Direct emissions

The fossil fuels associated with direct household emissions are gasoline, kerosene, liquefied petroleum gas (LPG), and city gas. As outlined in more detail below we convert the monthly household expenditures for these fuels elicited from the FIES, to g-CO_2_e. In summary, for each fuel, the household expenditures extracted from the FIES are first converted into corresponding mass or volume using retail fuel prices (see below). Fuel volume or mass is then converted into g-CO_2_e by multiplying with respective emission coefficients (see below).

Weekly retail prices for gasoline and kerosene come from a weekly survey conducted by the Ministry of Economy, Trade and Industry of Japan on retail prices at filling stations^47^. Monthly prices for gasoline and kerosene are obtained by averaging the prices of the weeks within each month. For kerosene, we adopt prices for on-site purchase, as the 2006 survey of kerosene and LPG consumption^48^ indicates that on-site purchases are be the principal means through which households purchase kerosene in Japan. For LPG, prices come from the monthly survey of retail prices conducted by the Oil Information Center, at the Institute of Energy Economics, Japan^49^. The LPG prices for the cities across the 47 prefectures are recorded as the overall prices of the corresponding geographical regions. As LPG retail prices are recorded in a stepwise manner for volumes (at 5 m^3^, 10 m^3^, 20 m^3^ and 50 m^3^), prefectural unit prices of LPG are set as the per unit price at one of the four recorded volumes just greater than the average volume purchased per month. Due to the lack of actual consumption data to distinguish the stepwise price window, we define this price window spatially using the most recent consumption data on LPG consumption in each of the 47 prefectures^50^. For instance, the average monthly consumption of LPG is 12.6 m^3^ per household in Chiba Prefecture. Therefore, the per cubic meter price of LPG (JPY/m^3^) purchased at 20 m^3^ is set to correspond to the per unit price for Chiba, as 20 m^3^ is the volume gradient that is immediately greater than 12.6 m^3^. Prices for city gas for the period 2011–2014 are captured by the Japanese Government Statistics^51^. As there is no data for the 2015 city gas price from the same data source we convert 2014 prices using Japan’s Consumer Price Index^52^.

Emission coefficients of gasoline, kerosene, LPG and city gas are provided by Japan’s Ministry of the Environment^53^. As LPG’s emission coefficient in the dataset is expressed with the unit tCO_2_/ton while LPG retail prices are expressed with the unit JPY/m^3^, a conversion is performed to convert the weight of liquid LPG (ton) into the volume of gaseous LPG (m^3^) using the conversion coefficient recommended by Ministry of the Environment^54^.

### Indirect emissions

The estimation of the indirect emissions embodied in the goods and services consumed by the household sector requires a cross-mapping and matching of the emission categories (and their intensities) from the 3EID dataset with the consumption categories of the FIES dataset.

The calculation of the indirect carbon emission intensity (*E*_*i*_) in the 3EID model is as follows:

1 $$\left(\begin{array}{c}{E}_{1}\\ \vdots \\ {E}_{k}\\ \vdots \\ {E}_{n}\end{array}\right)={\boldsymbol{D}}{({\boldsymbol{I}}-({\boldsymbol{I}}-\bar{{\boldsymbol{M}}}){\boldsymbol{A}})}^{-1}$$

where ***D*** is the direct emission matrix, ***I*** is the unit matrix, ${A}_{mn}=\frac{{x}_{mn}}{{x}_{m}}$ represents the output of industry *m* required to produce one unit of output from industry *n*, and $\bar{{\boldsymbol{M}}}$ is a diagonal matrix representing the import portion of the direct requirement coefficient. Due to its structure, the 3EID considers only domestic production (see “Limitations and Acknowledgements”). Further details of the input-output table and applications can be found elsewhere^10,37,55–60^.

As the classification of industries in the 3EID database differs from the classification of consumption elements in the FIES expenditure data, we matched the data following the general approach outlined elsewhere^33^, and outlined for the year 2015 in one of the supporting documents (see “Data Records”). In its emission intensity dataset, the 2011 3EID contains 395 items and the 2015 3EID contains 390 items. When cross-mapping the 3EID datasets with the corresponding FIES datasets, we end up with an emission inventory of 2011 to 2014 have 495 items for the period 2011–2014, and 512 items for the year 2015.

Even though the base data for the goods and services consumed by households that fall under indirect emissions are generated for each month between January 2011 and December 2015 under the FIES data (see “Dataset scope”), the indirect emission intensities relevant for each of these indirect emission categories after cross-mapping (see above) are generated only for the 2011 and 2015 input-output tables. This is because the 3EID databases that are used for the emission intensities are released every five years, and are thus only available for the years 2011 and 2015. To estimate the indirect emission intensities for each study items for the years 2012, 2013, and 2014 we use linear interpolation. Therefore, the values are obtained via an interpolation method as follows:

2 $$\left\{\begin{array}{c}{E}_{i}^{2012}=\frac{3}{4}{E}_{i}^{2011}+\frac{1}{4}{E}_{i}^{2015}\\ {E}_{i}^{2013}=\frac{1}{2}{E}_{i}^{2011}+\frac{1}{2}{E}_{i}^{2015}\\ {E}_{i}^{2014}=\frac{1}{4}{E}_{i}^{2011}+\frac{3}{4}{E}_{i}^{2015}\end{array}\right.$$

where ${E}_{i}^{j}$ indicates the carbon emission intensity of *i* in the year of *j*. ${E}_{i}^{2011}$ and ${E}_{i}^{2015}$ are generated from 3EID^43,44^, which respectively applied the 2011 and 2015 Japan input-output table.

It should be noted that electricity is treated as an indirect emission, as there is no direct emission upon its consumption (in contrast to the fossil fuels discussed in the previous section). Although the different cities contained in this dataset are supported from different electricity companies, we employ the national standard electricity intensity generated through the input-output tables.

It is worth noting that there is not always a perfect matching between (a) FIES and 3EID categories, and (b) within 2011 and 2015 3EID categories. These items are matched based on similarities in their properties. For (a) some 3EID categories such as waste management are not distinct household components in FIES. For this reason, they have been linked to relevant services items such as municipal services. However, some of the FIES miscellaneous expenses that are not expected to have indirect emissions such as allowances, grants for religious services, and donations have been omitted. For (b), some examples include categories in the 2011 3EID such as “small dried sardines”, “sewing machines” and “cloth tailoring” that do not have a perfect match in the 2015 3EID, and have been thus matched in similar categories such as “other salted food”, “consumer electrical equipment” and “other personal services” in 2015 3EID, respectively. This logic is also used when interpolating between years.

### Limitations and Acknowledgements

First, the FIES that is the base of the dataset used in this Data Descriptor does not cover single-person households. Single-person households are very prevalent in Japan^42^, and have very distinct consumption patterns, which often lead to higher per capita emissions in Japan^61^. This means that there is possibly an underestimation of the findings outlined in the “Technical Validation” section. Considering the comparatively high prevalence of single-person households in Japan^62^, there should be some caution or acknowledgement when using this dataset. It should be noted that similar to the FIES, the National Survey of Family Income and Expenditure (NSFIE)^63^ records the monthly consumption expenditures per household. Although the NSFIE covers a larger household sample than FIES (including single-person households), it does not record the consumption expenditures continuously. In particular the expenditures are based on a survey covering September to November for two- or more-person households, and one in November for single-person households. Furthermore, it is not conducted every year but every five years. Although this dataset has been utilized for household emissions at the city^64,65^, the fact remains that it has a lower periodicity and is not available publicly.

Second, the 3EID is an emission inventory generated through the Japanese single-regional input-output (SRIO) table. This means that the emission intensities used in this study reflect only domestic goods and services. By applying domestic emission intensities for imported goods, inserts uncertainty to the dataset, which is to a large degree unavoidable considering the lack of options to create a fine-grained dataset that also includes international emission intensities (see below). In particular, we select the 3EID, rather than a multi-regional input-output (MRIO) because of its higher sectoral resolution. In more detail, the 3EID has a much higher sectoral resolution (390 sectors), which brings it closer to the structure of the FIES that contains 500 consumption categories. This is a much more extensive coverage compared with other MRIOs such as WIOD (56 sectors) and EXIOBASE (200 sectors). This inability to consider properly emission intensities for imported goods and services might underestimate the actual GHG emissions for some consumption categories, as imported goods tend to have longer value chains, and thus higher GHG emissions when compared to similar domestic goods^24^. Regarding the regional heterogeneity, using a domestic subnational MRIO^66^ would be more preferable than 3EID cognizant of domestic regional heterogeneity^65^. However, there are no MRIOs covering recent time-series (e.g. 2011–2015) available yet.

Third, the latest available version of the 3EID input-output table covers emissions until the year 2015. Thus the dataset included in this Data Descriptor covers the period until December 2015. Furthermore, due to the use of city-scale statistics, it is not possible to disaggregate the dataset by income level or age group, which would have increased the explanatory power of the dataset.

## Data Records

The dataset contains monthly direct and indirect GHG emissions for 51–52 Japanese cities from 2011 to 2015. The direct emissions are recorded as Natural Gas, Gasoline, LPG, and Kerosene. The emissions are expressed in “per capita” terms. Overall, there is a total of 1,555,512 items, which include 1,543,128 items for indirect emissions and 12,384 items for direct emissions. Table 1 offers a summary of the data items for each study year.

**Table 1:** Data records for each study year.

Year	Cities	Months	Indirect emission items	Direct emission items	Indirect emission data records	Direct emission data records	Total data records
2011	51	12	495	4	302,940	2,448	305,388
2012	51	12	495	4	302,940	2,448	305,388
2013	52	12	495	4	302,940	2,496	311,376
2014	52	12	495	4	308,880	2,496	311,376
2015	52	12	512	4	319,488	2,496	321,984

The entire dataset is made public in Figshare, and is named “Monthly direct and indirect greenhouse gases emissions from household consumption in Japanese cities”^67^. It consists of 17 excel files (Table 2), which are explained in more detail below. For each study year, the data is included in two excel files, one for direct emissions (labeled as: “direct_20XX depending on the year) and one for indirect emissions (labeled as: “indirect_20XX depending on the year). Thus, the dataset spans a total of 10 excel files (Files 1–10) (Unit: g-CO_2_) The files for direct emissions, apart from the emission dataset itself, also contain the unit prices for direct energy consumption for each city for each month, provided by a separate file named Direct Emission Intensity.xlsx (Files 11–14). Here, direct emission intensity is provided in g-CO_2_/JPY, to facilitate future calculations on direct emission calculation at the city-scale (Files 11–14). Next, for reference (Files 15–16) we provide two excel files highlighting the cross-mapping of FIES and 3EID for the year 2015 (named Mapping.xlsx) and sector details (named FIES_items_Eng_2011-15.xlsx). Last, the household size information (File 17), includes total household size, children under 18 years old, aging above 65 years old, are given by a separate file named Household size information.xlsx.

**Table 2:** Description of dataset files.

File number	File name	File content
1	indirect_2011	Indirect emission for 2011
2	indirect_2012	Indirect emission for 2012
3	indirect_2013	Indirect emission for 2013
4	indirect_2014	Indirect emission for 2014
5	indirect_2015	Indirect emission for 2015
6	direct_2011	Direct emission for 2011
7	direct_2012	Direct emission for 2012
8	direct_2013	Direct emission for 2013
9	direct_2014	Direct emission for 2014
10	direct_2015	Direct emission for 2015
11	city_gas_intensity	Direct emission intensity for city gas for 2011–2015
12	gasoline_intensity	Direct emission intensity for gasoline for 2011–2015
13	kerosene_intensity	Direct emission intensity for kerosene for 2011–2015
14	lpg_intensity	Direct emission intensity for LPG for 2011–2015
15	Mapping	Cross-mapping of data items between FIES and 3EID dataset
16	FIES_items_Eng_2011–15	Names of FIES items
17	Household size information	Household information in terms of household member

## Technical Validation

Figure 2 shows the monthly average emissions of the cities included in this dataset for the period between 2011 and 2015. The results show that indirect emissions per capita are much higher than direct emissions per capita, accounting for 81.2% of total emissions. Naha and Sapporo are the lowest and highest per capita emitting cities, respectively. Lower per capita emitting cities are generally located in western Japan and Kyushu (e.g. Osaka, Fukuoka, Kumamoto, Nagasaki, Miyazaki and Naha). On the contrary, the higher per capita emitting cities are located in northeastern Japan (e.g. Sapporo and Akita). The cities in northeastern Japan have consistently higher direct and indirect emissions per capita compared to other cities in the country.

**Fig. 3:**
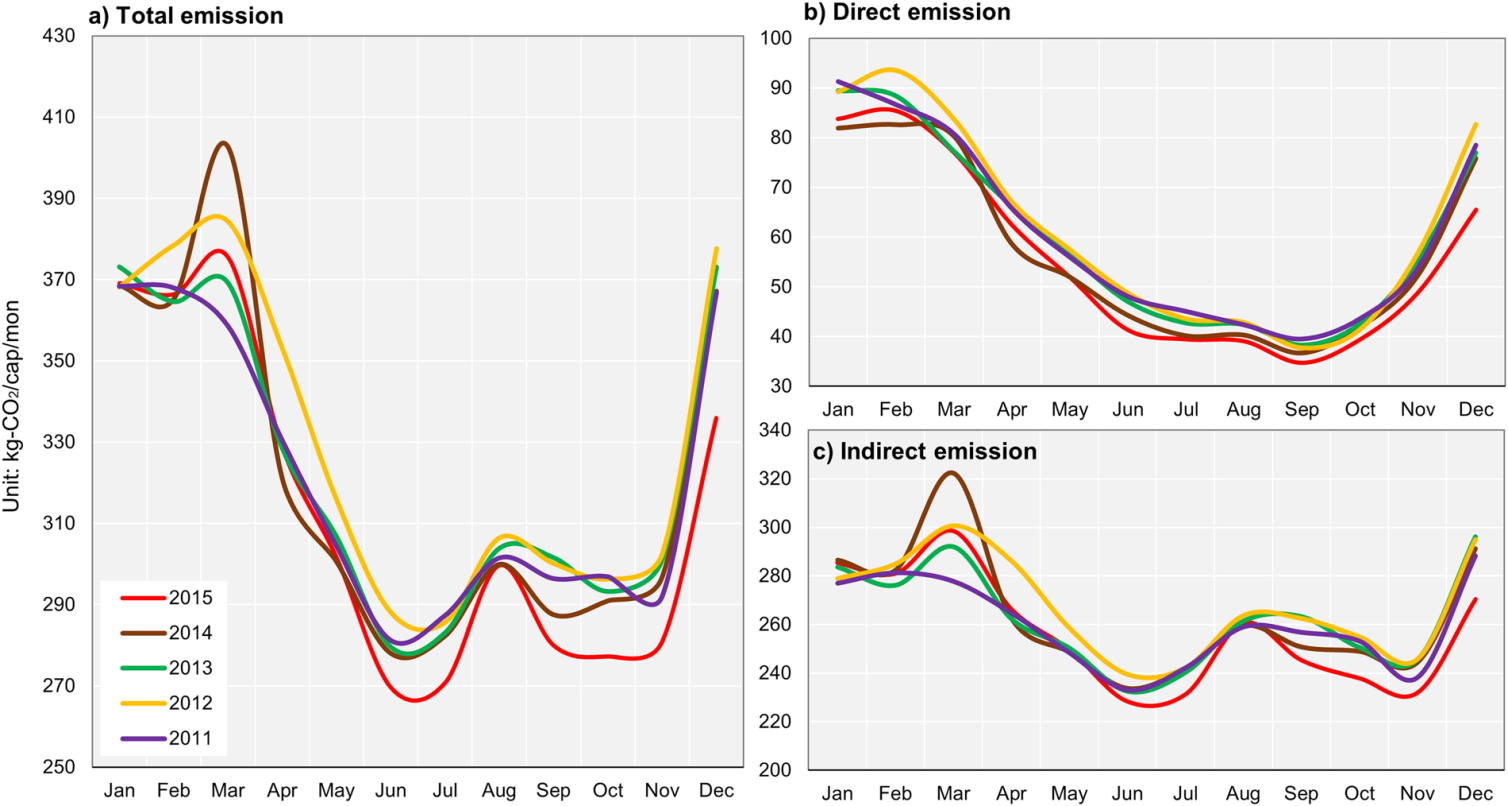
Monthly variation of GHG emissions averaged across cities.

**Fig. 4:**
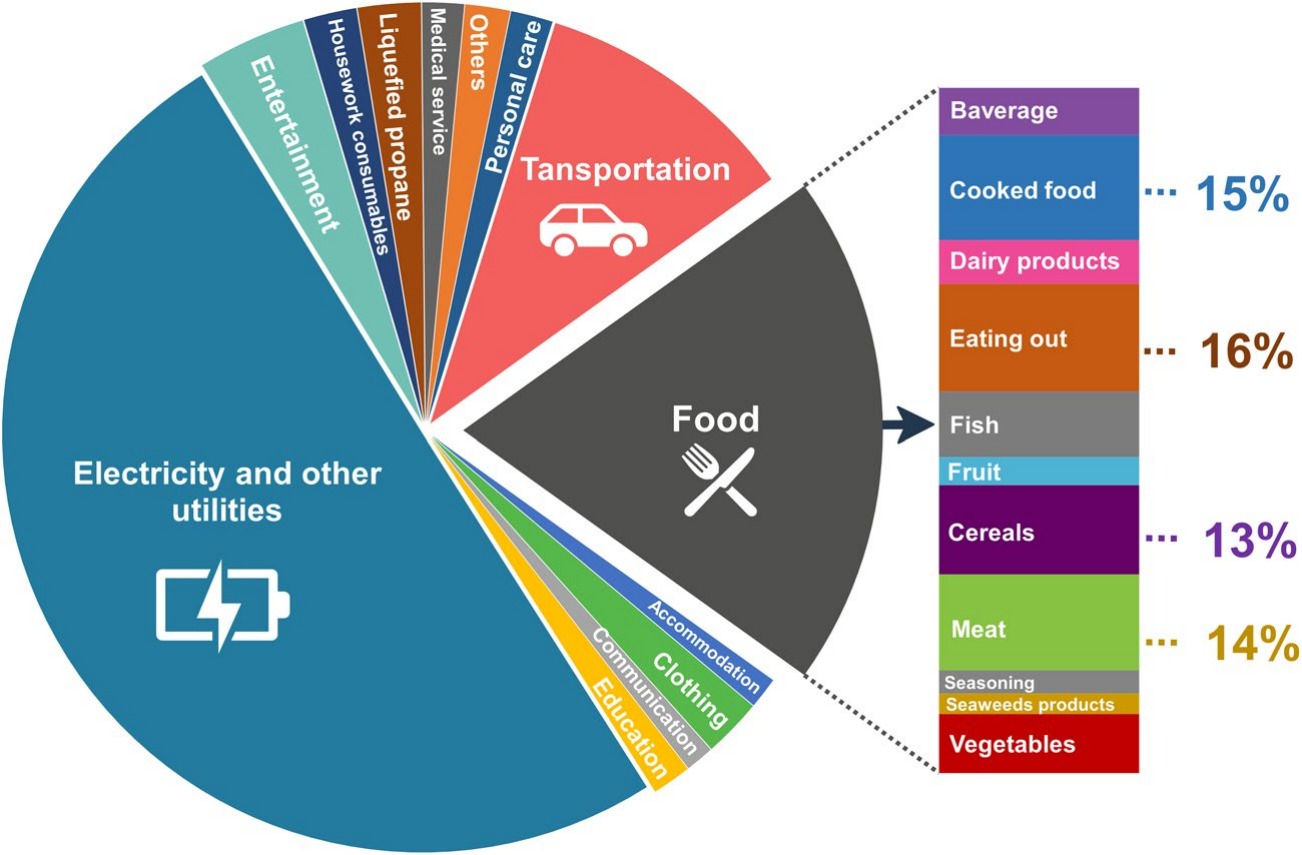
Breakdown of indirect emissions for 2015 across 512 consumption categories.

Figure 3 shows the monthly emission variation, averaged across cities, between 2011 and 2015. In particular, Fig. 3(a), shows the total emission averaged for all cities across years, and suggests higher total emissions for colder months such as December, January, and February. This trend is visible for both direct and indirect emissions, Fig. 3(b,c). However, two emission peaks are visible in March and August, which are mostly generated by indirect emissions. Figure 4 provides a simple break-down of indirect emissions averaged across 512 consumption elements for the year 2015. Approximately, 50.2% of total indirect emissions is due to electricity and other utilities, followed by food (19.8%). When disaggregating the food-related indirect emissions, emission from eating accounts for 16% of the total food-related emission, followed by processed food (15%), meat (14%), and cereals (13%) (Fig. 4**)**.

**Fig. 5:**
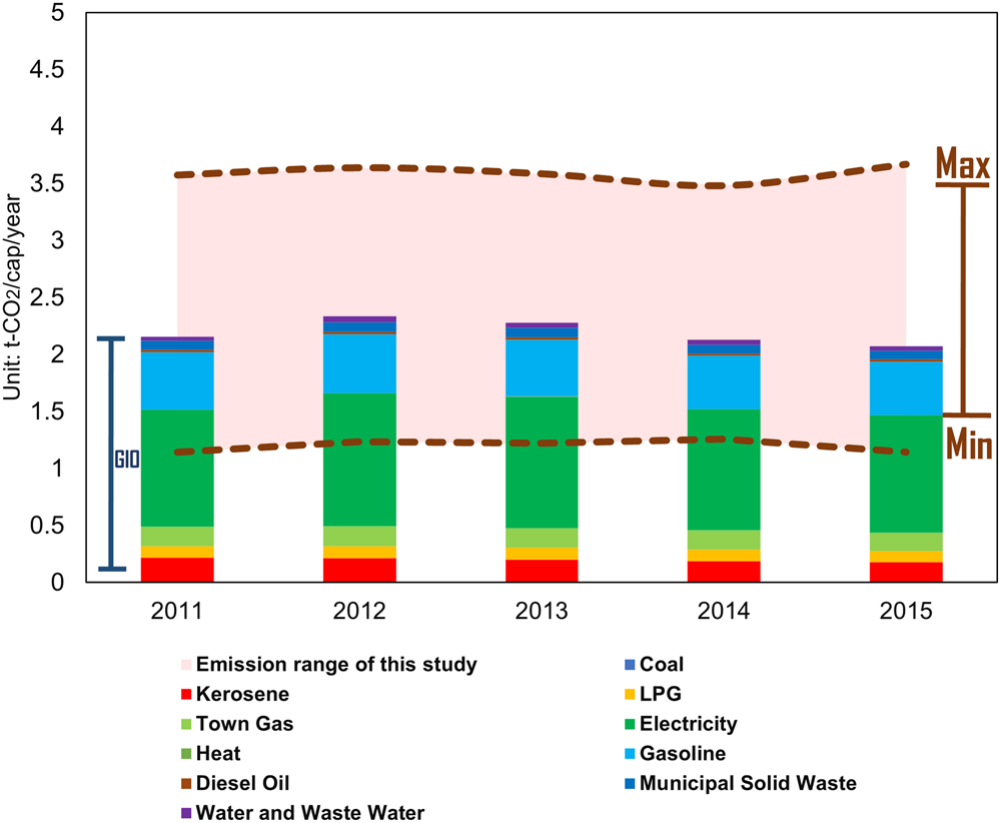
Comparison of direct emissions with GIO data.

Although there is no directly comparable dataset for validation purposes, we see a good correspondence of the direct emissions estimated through our dataset, with the relevant constituents of the Greenhouse Gas Inventory Office of Japan (GIO), at the National Institute for Environmental Studies (NIES) (Fig. 5). In Fig. 5 the pink shadow area shows the maximum and minimum value of our dataset (i.e. highest- and lowest-emitting cities), which falls within the average national estimates of GIO for direct emissions.

To note, for this validation we only use the direct emissions component of our dataset, since the indirect emissions are not available in any other currently available official statistics or other studies for validation purposes. However, we have to note that the indirect emission intensities in 3EID vary little over short timescales, such as the ones in this Data Descriptor (period between 2011–2015). For example, the emission intensities of rice in 2011 and 2015 are 8.1t-CO_2_e/M-JPY and 7.9 t-CO_2_e/M-JPY, respectively. Therefore, the variance is only 2.65% in a five-year period. Furthermore, the emission intensities for some durable goods changes even less, as production processes do not change dramatically. Thus we expect that the interpolation will not introduce major uncertainties in the calculation of indirect emissions for the years 2012, 2013 and 2014.

The authors of this Data Descriptor have used portions of this dataset to estimate the emissions of Japanese households for the years 2005 and 2011^33,68^. These previous studies have estimated for 2011 total emissions that range between 3.41–5.00 t-CO_2_e/cap, which are in accordance with the 2011 emission estimated through the current Data Descriptor. Total emission range between 2.98–4.39 t-CO_2_e/cap across 2011 to 2015, with cities’ average emission being 3.76, 3.86, 3.87, 3.96, and 3.85 t-CO_2_e/cap for each study year.

## Usage Notes

As cities have emerged as major actors in climate mitigation efforts in the past decades, there have been multiple initiatives to both estimate and quantify the contribution of cities to national and/or global emissions (see Background and Summary), as well as to develop city-level climate change mitigation strategies. For example, the Intergovernmental Panel on Climate Change (IPCC) 5^th^ Assessment Report pointed that progressive cities across the world have demonstrated significant political leadership by initiating meaningful strategies and actions to tackle climate change^69^. In this context the Urban Carbon Footprint (UCF) has been recognized as one of the more useful methodological options to inform decision-makers about environmental sustainability, both within and beyond city limits^70^.

That said, and mindful of its limitations (see Methods), this dataset can provide a very useful resource to urban researchers interested in analyzing different aspects of UCFs in a temporal and spatially differentiated manner. The data structure (i.e. direct and indirect emissions) and the method used to develop the emissions factors in this Data Descriptor have been discussed (and to some degree used) in previous UCF studies^33,68^. In this sense the method is rather universal in its approach.

However, what sets this Data Descriptor apart is the quality and comprehensiveness of the underlying data, both in relation to urban consumption (FIES data), as well as the emission intensity factors (3EID data). Thus, this dataset can allow the exploration of consumption patterns in a very disaggregated manner (>500 consumption items) and over different periods of time (i.e. monthly, annually). Due to data limitations at the urban scale, few studies have managed to calculate city-level household emission inventories in such a comprehensive manner. As outlined below, this dataset can appeal to researchers globally, as well as practitioners and policy-makers in the covered cities, and Japan more broadly.

In terms of research, some possible applications could be to identify, among others, (a) differences in the UCFs of cities^24,71^, (b) differences in consumption structure^21^, (c) differences in drivers of emissions by month, year, or city^33,72^, or (d) differences in emissions by income^26,73^, education^74,75^, or age^11,31,76^. Beyond city-level patterns, the dataset can be used to understand broader phenomena related to the environmental impacts of urban consumption. For example, the indirect emission component could be connected with other datasets focusing on specific demand to understand better emerging topics in urban studies such as urban tele-connections^77,78^, the transboundary environmental impacts of cities^79^, or inequalities in emissions^26,80^. Ideally this dataset can become an input to ongoing and future global reports on urban carbon mitigation, such as the reports prepared by the IPCC’s Working Group III.

In terms of policy and practice, the granular and location-specific data for various constituents of consumption can be used to identify potential priority areas for GHG emission reduction and facilitate better-informed and evidence-based mitigation actions by policy-makers in the covered cities. For example in some cities in northern Japan such as Sapporo and Aomori, kerosene consumption accounts for a large percentage of the direct GHG emissions. This suggests that the electrification of heating could be an important GHG mitigation measure^33,68^. By providing the emission inventories for each month, it is possible to facilitate the understanding of seasonal emission patterns, providing an even finer print of household emissions, and the development of decarbonization measures through behavioral change. Furthermore, this dataset can be used to explore differentiated emission profiles across households with different characteristics such as income, age or education. By identifying better the different emission profiles of such groups it can help city governments create more nuanced and targeted measures to affect consumption and emission behavior across different types of households or investigate decarbonization scenarios in a more nuanced way. The above could inform the generation of good practices for how to use such high-resolution data to track household carbon footprints according to daily consumption behaviors, which can possibly be applied in other urban contexts around the world.

## Data Availability

No code was used in the generation of the data.
